# Case Report: Recurrent retinal vein occlusion as the first clinical manifestation of systemic lupus erythematosus in a male patient

**DOI:** 10.12688/f1000research.55189.3

**Published:** 2022-01-10

**Authors:** Marwa Ben Brahim, Sondes Arfa, Fadia Boubaker, Jihen Chelly, Wafa Ammari, Sonia Hammami, Fatma Arbi, Olfa Berriche

**Affiliations:** 1 Department of Internal Medicine and Endocrinology, Taher Sfar University Hospital, University of Monastir, Mahdia, 5100, Tunisia; 2 Department of Ophthalmology, Taher Sfar University Hospital, University of Monastir, Mahdia, 5100, Tunisia; 3 Department of Internal Medicine and Endocrinology, Fattouma Bourguiba University Hospital, University of Monastir, Monastir, 5000, Tunisia; 4 Biochemistry Laboratory,LR12ES05 LR-NAFS Nutrition-Functional Food and Vascular Health, Faculty of Medicine, University of Monastir, Monastir, 5000, Tunisia

**Keywords:** Retinal vein occlusion, Systemic Lupus Erythematosus, Male patient, Intra-vitreal anti-vascular endothelial growth factor antibodies treatment, case report

## Abstract

Systemic lupus erythematosus (SLE) is a chronic, autoimmune disease characterized by widespread clinical manifestations and immunological disorders. A myriad of ocular manifestations can be seen in patients with SLE. The most vision-threatening complication is vaso-occlusive retinopathy including retinal vein occlusion (RVO). RVO associated with SLE is well described in the literature and its association with antiphospholipid antibodies is recognized. However, RVO as the initial manifestation of SLE is scarcely reported.

Herein, we report the first case of recurrent RVO as the revealing manifestation of SLE in a 40-year-old male patient. He had two consecutive episodes of decreased vision. Ophthalmologic examination disclosed a branch retinal vein occlusion the first time and a central retinal vein occlusion the second time. The diagnosis of SLE was established based on clinical and immunological criteria. He was prescribed antiplatelet therapy, hydroxychloroquine at 5.5 mg/kg/day, and intravitreal anti-vascular endothelial growth factor (VEGF) antibodies regimen. He slowly improved under treatment.

## Introduction

Retinal vein occlusion (RVO) is a common retinal vascular disorder that, if left untreated, can lead to vision loss. ^1^ Classic risk factors are hypertension, hyperlipidemia and diabetes mellitus. ^2^ Systemic and inflammatory diseases such as systemic lupus erythematosus (SLE) and antiphospholipid syndrome were found to be associated with the development of RVO. ^2^ RVO associated with SLE is well described in the literature and its association with antiphospholipid antibodies is recognized. ^1, 2^ However, RVO as the initial manifestation of SLE is very uncommon. Herein we report a unique case of recurrent RVO as the initial presentation of SLE in a male patient.

## Case report

A 40-year-old Caucasian man, with no family history of autoimmune diseases and a personal medical history of hypertension, was admitted to the Ophthalmology Department of Taher Sfar University Hospital with blurred vision in the right eye. On detailed physical examination, he had no fever, arthritis, or chest complaints. On ophthalmologic examination, the best corrected visual acuity was 20/20, and a retinal branch vein occlusion in the right eye was disclosed. He was treated with aspirin (100 mg/day) associated with equilibration of his hypertension.

**Figure 1.**
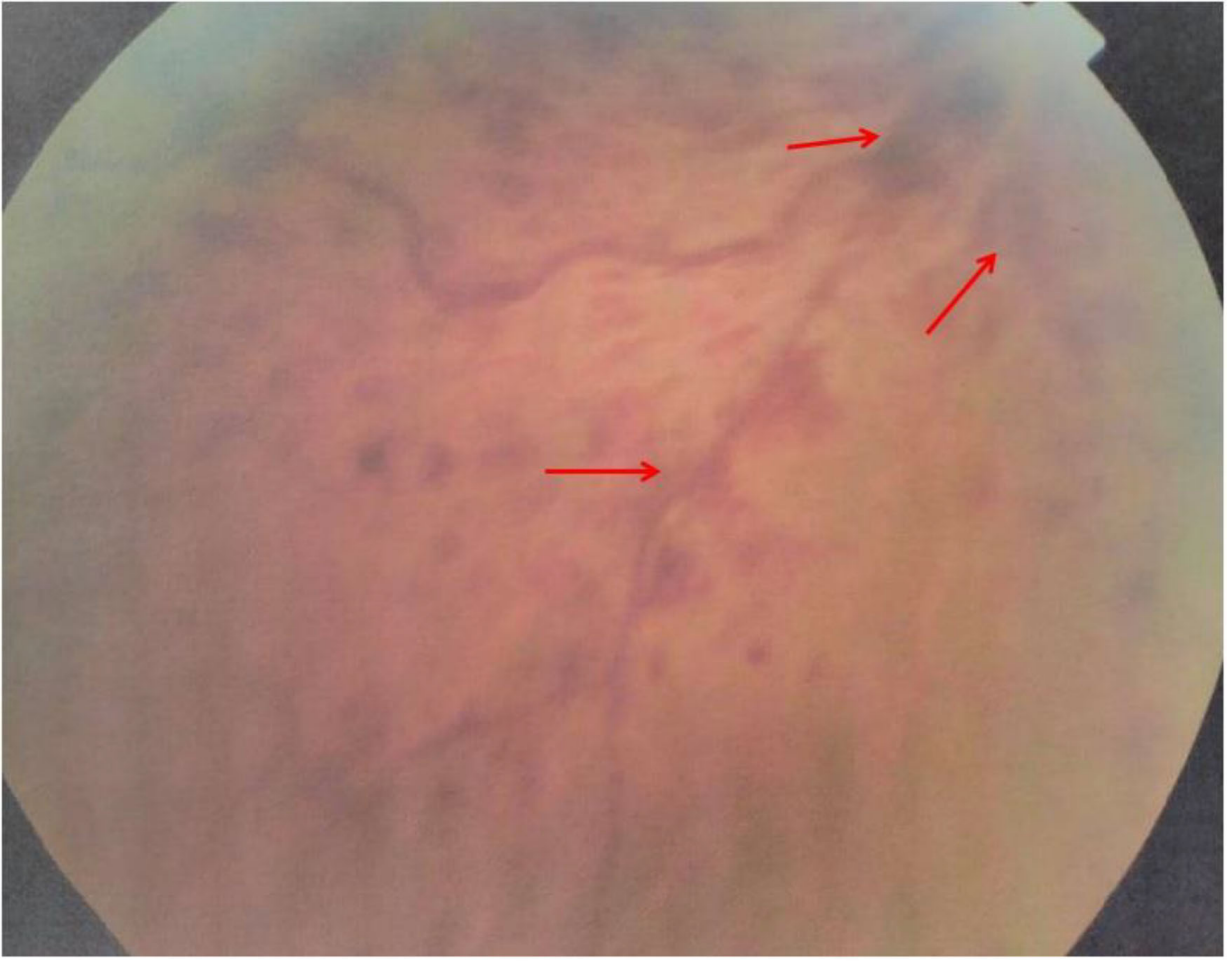
Superficial flame-shaped retinal hemorrhages in fundus examination.

**Figure 2.**
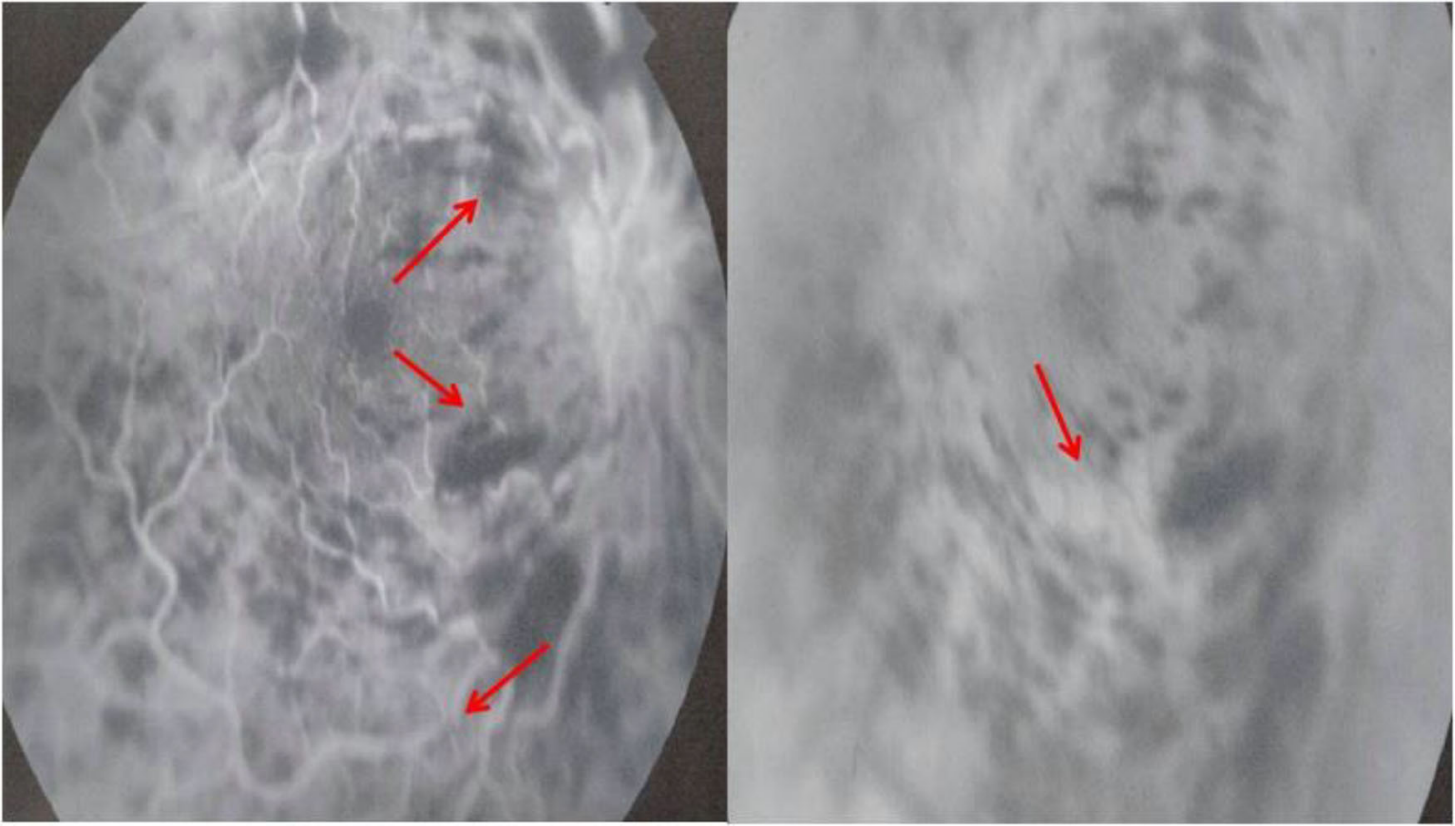
Fluorescein angiography showing vascular tortuosity, retinal hemorrhage, and cotton wool spots on the right eye.

**Figure 3.**
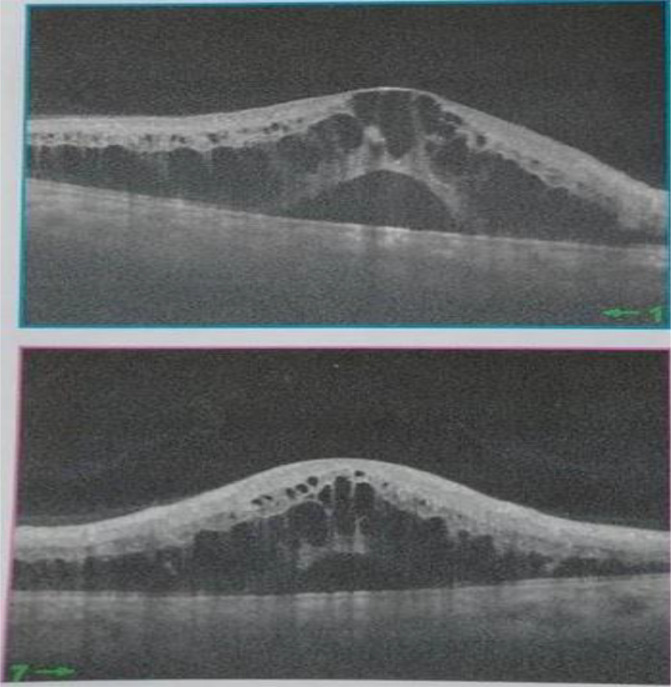
Spectral-domain optical coherence tomography showing cystoid macular oedema.

One year later, he experienced another episode of blurred and decreased vision in the same eye. Physical examination was unremarkable. A skin exam revealed he had an erythema over the malar area. His blood pressure was normal. Fundus examination disclosed central retinal vein occlusion, superficial flame-shaped retinal hemorrhages, and macular oedema ( Figure 1). Fluorescein angiography (FA) demonstrated vascular tortuosity, retinal hemorrhage, and cotton wool spots on the right eye ( Figure 2). Spectral-domain optical coherence tomography demonstrated cystoid macular oedema ( Figure 3). The left eye examination showed normal sizes of the retinal vessels and retina. A refraction study showed a best corrected visual acuity at 20/70 in the right eye and 20/20 in the left eye. On laboratory investigations, a blood test showed platelets: 229 * 10 ^9^/l, leukocytes: 9 * 10 ^9^/l, and hemoglobin level: 13.5 g/dl. Erythrocyte sedimentation rate was 30.

Autoantibodies tests revealed positive antinuclear antibodies (1: 800), anti-DNA antibodies, anti-nucleosomes antibodies, and slightly positive anti-citrullinated protein antibodies and rheumatoid factors. Antiphospholipid antibodies screening displayed high titer (> 40 UI) of IgG anticardiolipines and IgG antiβ2 glycoprotein antibodies. Total blood complement, C3, C4, protein S, protein C and antithrombin III levels were normal. The diagnosis of SLE was established based on clinical and immunological criteria including malar rash, positive anti-nuclear antibodies, anti-DNA antibodies, and antiphospholipid antibodies.

The patient was started with hydroxychloroquine at 5.5 mg/kg/day and intra-vitreal anti-vascular endothelial growth factor (VEGF) antibodies regimen, in combination with aspirin (100 mg/day). The patient is still regularly taking his treatment without significant side effects. His vision has slowly improved under treatment. The patient remained under close observation. After two years of follow up, a refraction study showed a stable visual acuity.

## Discussion

The atypical clinical presentation of SLE, in a male patient with a medical history of hypertension, and without any clinical objective criteria, led to the delay of the diagnosis of this autoimmune disease. The diagnosis was made after a second retinal vein occlusion. The patient had cutaneous involvement concomitantly with ocular complication. He had immunological criteria including positive antinuclear antibodies, anti-DNA antibodies and antiphospholipid antibodies which made the diagnosis clearer.

SLE is a chronic and autoimmune disease characterized by widespread clinical manifestations and immunological disorders. It occurs in both genders but it is much more common in females than males, with female:male sex-ratio of 8:1 to 15:1. ^3^ Male patients have a higher prevalence of life threatening manifestations including lupus nephritis, central neurological system involvement and hemolytic anemia. ^3, 4^ Regarding immunological features, anti-phospholipid antibodies were found to be more frequent in male SLE patients. ^5^ Thus, it would be expected that they present an increased risk of thromboembolic manifestations and antiphospholipid syndrome, which could worsen the course of the illness and increase the mortality rates. We report a case of SLE associated with antiphospholipid antibodies in a male patient. He presented a recurrent RVO as the first manifestation of the disease making this case unique.

A myriad of ocular manifestations can be seen in patients with SLE including keratoconjunctivitis, scleritis, episcleritis, retinopathy, choroidopathy, orbital and lachrymal system disorders. ^6^ The most common ocular manifestation is keratoconjunctivitis but the most visually-threatening is retinopathy. The prevalence of lupus retinopathy varies from 3% to 28%. ^6, 7^ The most common manifestations of lupus retinopathy are cotton wool spots, retinal hemorrhage and optic disk oedema. ^7^ Vaso-occlusive retinopathies is a subset of retinal vasculopathy, including retinal artery or vein occlusions which are a rare but severe complication. The vascular retinopathy in SLE results from immune complex mediated vascular injuries and micro-vascular thrombosis. ^8^ Patients with retinal vessel occlusion seem to have a poorer visual prognosis.

Patients with SLE have a higher prevalence of developing RVO than the general population. A higher incidence of antiphospholipid antibodies in SLE patients with RVO has been reported. ^7, 9, 10^ However, the patient had a slightly positive anti-citrullinated protein antibodies and rheumatoid factor without bone erosion or joint stiffness or deformity evoking the diagnosis of rhupus. In fact, positivity of anti-CCP can be seen in 10-15% of patients with SLE without an association to rheumatoid arthritis. ^11^ Typically, RVO occurs in the first four years follow-up of SLE. Retinal vasculitis was scarcely reported as the first manifestation of SLE. ^12– 14^ As far as we know, this would be the first case of a recurrent RVO as the revealing presentation of SLE to be reported in literature.

Regarding the treatment of RVO in patients with SLE, anticoagulation and anti-platelet therapies have contributed to the stabilization of the retinal occlusion and the prevention of recurrent thrombosis either used separately or combined. The use of an immunosuppressant is still controversial due to the lack of evidence about its effects in improving the visual acuity and the retinal vascular occlusion recurring. ^7^ Intravitreally administrated anti-VEGF antibodies were introduced in the treatment regimen of RVO. Its main desired effect is to reduce the macular edema, which is the major cause of decreased visual acuity in patients with RVO. ^15^ Our patient received a combination of anti-platelet therapy and anti-VEGF antibodies. Clinical improvement was achieved under this treatment.

## Conclusion

SLE in males may have an atypical presentation. This often leads to a delay in making the diagnosis and starting treatment. In this article, we have reported a unique case of SLE in a male patient presenting with a severe and sight- threatening ocular complication. The diagnosis was overlooked, as the patient did not have any clinical criteria of SLE initially. Our case report’s core contribution is to raise awareness about the possible typical and severe presentation of SLE in men.

## Consent

Written informed consent for publication of their clinical details and clinical images was obtained from the patient.

## Data availability statement

All data underlying the results are available as part of the article and no additional source data are required.

## References

[1] Jaulim A Ahmed B Khanam T : Branch retinal vein occlusion: epidemiology, pathogenesis, risk factors, clinical features, diagnosis, and complications. An update of the literature. *Retina Phila Pa.* 2013;33(5):901–10. 10.1097/IAE.0b013e3182870c15 23609064

[2] Hernández JL Sanlés I Pérez-Montes R : Antiphospholipid syndrome and antiphospholipid antibody profile in patients with retinal vein occlusion. *Thromb Res.* 2020;190:63–8. 10.1016/j.thromres.2020.04.005 32311631

[3] Pons-Estel GJ Ugarte-Gil MF Alarcón GS : Epidemiology of systemic lupus erythematosus. *Expert Rev Clin Immunol.* 2017;13(8):799–14. 10.1080/1744666X.2017.1327352 28471259

[4] do Socorro Teixeira Moreira Almeida M da Costa Arcoverde J Barros Jacobino MN : Male systemic lupus erythematosus, an overlooked diagnosis. *Clin Pract.* 2011;e103:1. 10.4081/cp.2011.e103 24765344 PMC3981404

[5] Dey D Ofori E Hutton-Mensah KA : Clinical characteristics of males with systemic lupus erythematosus (SLE) in an inception cohort of patients in Ghana. *Ghana Med J.* 2019;53(1):2–7. 10.4314/gmj.v53i1.1 31138937 PMC6527833

[6] Silpa-archa S Lee JJ Foster CS : Ocular manifestations in systemic lupus erythematosus. *Br J Ophthalmol.* 2016;100(1):135–41. 10.1155/2012/290898 25904124

[7] Au A O’Day J : Review of severe vaso-occlusive retinopathy in systemic lupus erythematosus and the antiphospholipid syndrome: associations, visual outcomes, complications and treatment. *Clin Experiment Ophthalmol.* 2004;32(1):87–100. 10.1046/j.1442-9071.2004.00766.x 14746601

[8] Yen Y-C Weng S-F Chen H-A : Risk of retinal vein occlusion in patients with systemic lupus erythematosus: a population-based cohort study. *Br J Ophthalmol.* 2013;97(9):1192–6. 10.1136/bjophthalmol-2013-303265 23832964

[9] Montehermoso A Cervera R Font J : Association of antiphospholipid antibodies with retinal vascular disease in systemic lupus erythematosus. *Semin Arthritis Rheum.* 1999;28(5):326–32. 10.1016/s0049-0172(99)80017-1 10342390

[10] Nangia PV Viswanathan L Kharel R : Retinal Involvement in Systemic Lupus Erythematosus. *Lupus Open Access.* 2017;2:1000129. 10.35248/2684-1630.17.2.129

[11] Kakumanu P Sobel ES Narain S : Citrulline dependence of anti-cyclic citrullinated peptide antibodies in systemic lupus erythematosus as a marker of deforming/erosive arthritis *J Rheumatol.* 2009;36(12):2682–90. 19884269 10.3899/jrheum.090338 PMC2803319

[12] Alhassan E Gendelman HK Sabha M : Bilateral Retinal Vasculitis as the First Presentation of Systemic Lupus Erythematosus. *Am J Case Rep.* 2021;22:e930650. 10.12659/AJCR.930650 33935278 PMC8105742

[13] Bandyopadhyay SK Moulick A Dutta A : Retinal vasculitis--an initial presentation of systemic lupus erythematosus. *J Indian Med Assoc.* 2006;104(9):526–7. 17388013

[14] Kremer I Gilad E Cohen S : Combined arterial and venous retinal occlusion as a presenting sign of systemic lupus erythematosus. *Ophthalmol J Int Ophtalmol Int J Ophthalmol Z Augenheilkd.* 1985;191(2):114–8. 10.1159/000309570 4058856

[15] Stahl A Agostini H Hansen LL : Bevacizumab in retinal vein occlusion-results of a prospective case series. *Graefes Arch Clin Exp Ophthalmol.* 2007;245(10):1429–36. 10.1007/s00417-007-0569-6 17356824

