# Supplementary material for: Case Report: Recurrent retinal vein occlusion as the first clinical manifestation of systemic lupus erythematosus in a male patient
Source: F1000Res. 2022 Jun 22;10:761. Originally published 2021 Aug 5. [Version 4] doi: 10.12688/f1000research.55189.4 (PMC9226707; doi:10.12688/f1000research.55189.4)
Supplement: Peer review history

# Peer review: Reviewer response for version 4

**DOI:** 10.5256/f1000research.135108.r141742

**Published:** 2022-06-23

**Authors:** Matias Iglicki

**Affiliations:** 1 Private Retina Practice, University of Buenos Aires, Buenos Aires, Argentina

Authors have addressed the questions properly.

Are enough details provided of any physical examination and diagnostic tests, treatment given and outcomes?

Yes

Is the case presented with sufficient detail to be useful for other practitioners?

Partly

Is sufficient discussion included of the importance of the findings and their relevance to future understanding of disease processes, diagnosis or treatment?

Partly

Is the background of the case’s history and progression described in sufficient detail?

Partly

Reviewer Expertise:

DME, RVO, NAMD and retina surgical cases

I confirm that I have read this submission and believe that I have an appropriate level of expertise to confirm that it is of an acceptable scientific standard.

# Peer review: Reviewer response for version 3

**DOI:** 10.5256/f1000research.84622.r119240

**Published:** 2022-02-09

**Authors:** Bouomrani Salem

**Affiliations:** 1 Sfax Faculty of Medicine, University of Sfax, Sfax, 3029, Tunisia; 2 Department of Internal medicine, Military Hospital of Gabes, Gabes, 6000, Tunisia

The authors have modified the manuscript as requested. This version of the manuscript can be indexed.

Are enough details provided of any physical examination and diagnostic tests, treatment given and outcomes?

Partly

Is the case presented with sufficient detail to be useful for other practitioners?

Yes

Is sufficient discussion included of the importance of the findings and their relevance to future understanding of disease processes, diagnosis or treatment?

Yes

Is the background of the case’s history and progression described in sufficient detail?

Partly

Reviewer Expertise:

Connective tissue disease, autoimmunity, immunogenic.

I confirm that I have read this submission and believe that I have an appropriate level of expertise to confirm that it is of an acceptable scientific standard.

# Peer review: Reviewer response for version 2

**DOI:** 10.5256/f1000research.78021.r96914

**Published:** 2021-12-06

**Authors:** Bouomrani Salem

**Affiliations:** 1 Sfax Faculty of Medicine, University of Sfax, Sfax, 3029, Tunisia; 2 Department of Internal medicine, Military Hospital of Gabes, Gabes, 6000, Tunisia

- The corrections requested for the "Discussion" section have not been made.
- Similarly, the suggested bibliographical references, dealing with the subject of this paper and very useful for enriching the discussion, have not been used.

This version should be revised in accordance with these suggestions.

Are enough details provided of any physical examination and diagnostic tests, treatment given and outcomes?

Partly

Is the case presented with sufficient detail to be useful for other practitioners?

Yes

Is sufficient discussion included of the importance of the findings and their relevance to future understanding of disease processes, diagnosis or treatment?

Yes

Is the background of the case’s history and progression described in sufficient detail?

Partly

Reviewer Expertise:

Connective tissue disease, autoimmunity, immunogenic.

I confirm that I have read this submission and believe that I have an appropriate level of expertise to confirm that it is of an acceptable scientific standard, however I have significant reservations, as outlined above.

# Response

**Published:** 2021-12-14

**Authors:** Marwa Ben Brahim

**Affiliations:** Taher Sfar University Hospital, University of Monastir, Tunisia

Dear Reviewer;

 The requested corrections for the discussion section are now made as you previously suggested.

# Peer review: Reviewer response for version 1

**DOI:** 10.5256/f1000research.58746.r91273

**Published:** 2021-09-23

**Authors:** Matias Iglicki

**Affiliations:** 1 Private Retina Practice, University of Buenos Aires, Buenos Aires, Argentina

Marwa Ben Brahim *et al. *present an interesting study about Retinal vein occlusion (RVO) as the first clinical manifestation of systemic lupus erythematosus (SLE) in a male patient. The study results certainly suggest to some degree that the RVO as the initial manifestation of SLE is scarcely reported.

 Besides how the magnitude of these data add new findings compare to the current standard can not be determined only based on this study. The results are encouraging and further study is warranted.

 Here some relevant points:

1. Please add on keywords - these do not match with the manuscript.
2. The authors should express why it is relevant for an RVO patient to link Retinal vein occlusion as the first clinical manifestation of systemic lupus erythematosus in a male patient? What does it change for the current standard of care?
3. The authors should explain why their findings make a difference for ophthalmologists around the world and for the readers of *F1000Research.*
4. The authors should explain the source of the information and what criteria they used for adding it to the paper. Were the assessors masked? What was the ICC (Inter class Correlation) between them in order to analyze the data? Was the randomization digitalized?
5. Please add in the introduction that papers have been published showing how the Optical Coherence Tomography (OCT) and new devices lead us to proper diagnoses in Retinal diseases - add one line in the introduction of this and also in the discussion section. These papers should be described in the general considerations. See references ^1^^,^^2^^,^^3^. 
6. Please add how and how long takes for a retina specialist to link and ask the patient about SLE and other Rheumatology and Rheumatic Diseases.
7. Results could be misinterpreted - add a short summary of the similarities in different devices and also add different OCT modalities, etc., and what can be improved in the process of detecting RVO is mandatory in the discussion section i.e wide-field angiography, different types of OCT modalities OCT angiography (OCTa).
8. Please apply correction for misspelling and English grammar.

Are enough details provided of any physical examination and diagnostic tests, treatment given and outcomes?

Yes

Is the case presented with sufficient detail to be useful for other practitioners?

Partly

Is sufficient discussion included of the importance of the findings and their relevance to future understanding of disease processes, diagnosis or treatment?

Partly

Is the background of the case’s history and progression described in sufficient detail?

Partly

Reviewer Expertise:

DME, RVO, NAMD and retina surgical cases

I confirm that I have read this submission and believe that I have an appropriate level of expertise to confirm that it is of an acceptable scientific standard, however I have significant reservations, as outlined above.

## References

[1] : UNDERDIAGNOSED OPTIC DISK PIT MACULOPATHY: Spectral Domain Optical Coherence Tomography Features For Accurate Diagnosis. *Retina* .2019;39(11) : 10.1097/IAE.0000000000002270 2161-2166 10.1097/IAE.0000000000002270 30045135

[2] : Effectiveness and Safety of Intravitreal Dexamethasone Implant (Ozurdex) in Patients with Diabetic Macular Edema: A Real-World Experience. *Ophthalmologica* .2019;241(1) : 10.1159/000492132 9-16 10.1159/000492132 30408801

[3] : TRActional DIabetic reTInal detachment surgery with co-adjuvant intravitreal dexamethasONe implant: the TRADITION STUDY. *Acta Diabetol* .2019;56(10) : 10.1007/s00592-019-01357-y 1141-1147 10.1007/s00592-019-01357-y 31089929

# Response

**Published:** 2022-06-13

**Authors:** Marwa Ben Brahim

**Affiliations:** Taher Sfar University Hospital, University of Monastir, Tunisia

1. Keywords: they match the manuscript
2. Since systemic lupus erythematosus is a chronic inflammatory disease with a periodic evolution it may require anti-inflammatory and/or immunosuppressant treatments in addition to symptomatic treatments of visceral manifestation such as the case of RVO how required Hydroxychloroquine, aspirin, and antiangiogenic treatment as well as long term follow-up in order to reduce relapses.
3. In fact, publishing this case report may raise awareness about this severe sight-threatening manifestation of SLE in men patients.
4. Dear reviewer, I do not think that interclass correlation could be applied in a case report.
5. Optical coherence tomography (OCT) allows the visualization of retinal layers and early diagnosis of the small retinal lesions.
6. Although the scarcity of ophthalmological manifestations as the primary manifestation of SLE, retina specialists should keep this diagnosis in mind and carefully look for other signs of an autoimmune systemic disease.
7. Done.

# Peer review: Reviewer response for version 1

**DOI:** 10.5256/f1000research.58746.r91272

**Published:** 2021-08-17

**Authors:** Bouomrani Salem

**Affiliations:** 1 Sfax Faculty of Medicine, University of Sfax, Sfax, 3029, Tunisia; 2 Department of Internal medicine, Military Hospital of Gabes, Gabes, 6000, Tunisia

The authors provide an interesting and original Case Report of retinal venous occlusion revealing systemic lupus erythematosus in a man. This observation is distinguished by the initial atypical clinical manifestation, male sex, and recurrence of retinal occlusion. This case is therefore the first to report recurrent retinal venous occlusion as an initial manifestation of lupus.

 Some comments are however useful to improve the quality of this manuscript:

 1. Title:

- Replace the proposed title with "recurrent retinal vein occlusion as..." to highlight the recurrent character which is the originality of this observation.

2. Abstract:

- Replace “primary” by initial or revealing.

3. Keywords:

- Adapt the list of keywords to international standards: remove “patient” from “male patient”, remove “case report”, remove “intra- vitreal and treatment” from “Intra-vitreal anti-vascular endothelial growth factor antibodies treatment”.

4. Case Report:

- Specify the anti-hypertensive treatment received by the patient (possibility of induced lupus)?.
- Replace "caucasian" by "Tunisian".
- Remove "s" from "rheumatoid factors",
- Add, if possible, a photo of the patient's malar erythema.
- If possible, give the results of the explorations made to support the diagnosis of systemic lupus erythematosus: cardiac ultrasound (lupus pericarditis? which is asymptomatic in 30% of cases), cerebral MRI (infra-clinical neurolupus? Especially the association with retinal vasculitis is noted in more than 70% of cases ^1^), and urinalysis?.
- Specify, if they were carried out, the results of the following tests: factor V mutation? (main thrombophilia causing venous thrombosis in Tunisia), and homocysteinemia? (hyperhomocysteinemia may be an added risk factor for retinal vein occlusions during SLE (1 patient/3 in Kumar K *et al* series ^2^).

5. Discussion:

- Replace "primary" by initial or revealing.
- Discuss the significance of anti-CCP antibodies in this observation: associated rheumatoid arthritis? (positive anti-CCP antibodies and positive RF: Rhupus syndrome?) Or a simple positivity of anti-CCP which can be seen in 10-15% of patients with SLE ^3^?

6. Conclusion:

- Rephrase “delay in making the diagnosis…” by “diagnostic and therapeutic delay”.
- Replace "article" by "paper".
- Correct "typical" by "atypical".
- Emphasize the recurrent and revealing nature of the retinal venous occlusion which is the originality of this case.

Are enough details provided of any physical examination and diagnostic tests, treatment given and outcomes?

Partly

Is the case presented with sufficient detail to be useful for other practitioners?

Yes

Is sufficient discussion included of the importance of the findings and their relevance to future understanding of disease processes, diagnosis or treatment?

Yes

Is the background of the case’s history and progression described in sufficient detail?

Partly

Reviewer Expertise:

Connective tissue disease, autoimmunity, immunogenic.

I confirm that I have read this submission and believe that I have an appropriate level of expertise to confirm that it is of an acceptable scientific standard, however I have significant reservations, as outlined above.

## References

[1] : Systemic lupus erythematosus presenting earlier as retinal vaso-occlusion. *Korean J Intern Med* .2001;16(3) : 10.3904/kjim.2001.16.3.210 210-3 10.3904/kjim.2001.16.3.210 11769581PMC4531720

[2] : Severe Vaso-Occlusive Retinopathy in Systemic Lupus Erythematosus: A Case Series. *Cureus* .2021; 10.7759/cureus.13019 10.7759/cureus.13019 PMC792079233665046

[3] : Citrulline dependence of anti-cyclic citrullinated peptide antibodies in systemic lupus erythematosus as a marker of deforming/erosive arthritis. *J Rheumatol* .2009;36(12) : 10.3899/jrheum.090338 2682-90 10.3899/jrheum.090338 19884269PMC2803319

# Response

**Published:** 2021-09-27

**Authors:** Marwa Ben Brahim

**Affiliations:** Taher Sfar University Hospital, University of Monastir, Tunisia

1. Done

 2. Done

 3. Done, the keyword "case report" was kept because it has to be mentioned.

 4. We, unfortunately, do not have any pictures of the patient's malar rash. 

 Since the patient had not had any neurological symptoms, we did not demand a Brain-MRI.

 We have assed further details about the laboratory investigation.

 5. Done. The patient had slightly positive anti-citrullinated protein antibodies and rheumatoid factor without bone erosion or joint stiffness or deformity evoking the diagnosis of rhupus. In fact, the positivity of anti-CCP can be seen in 10-15% of patients with SLE without an association to rheumatoid arthritis.

 6. We rephrased "delay in making the...." as you suggested. We corrected typical by atypical as you suggested. We have emphasized the recurrent and revealing character of the retinal vein occlusion in all parts of the paper.
